# Knowledge, Attitudes and Practices (KAP) related to the Pandemic (H1N1) 2009 among Chinese General Population: a Telephone Survey

**DOI:** 10.1186/1471-2334-11-128

**Published:** 2011-05-16

**Authors:** Yilan Lin, Lijuan Huang, Shaofa Nie, Zengyan Liu, Hongjie Yu, Weirong Yan, Yihua Xu

**Affiliations:** 1 Department of Epidemiology and Biostatistics, School of Public Health, Tongji Medical College, Huazhong University of Science and Technology, Wuhan 430030, China; 2 Office for Disease Control and Emergency Response, Chinese Centre for Disease Control and Prevention (CDC), Beijing 10013, China

## Abstract

**Background:**

China is at greatest risk of the Pandemic (H1N1) 2009 due to its huge population and high residential density. The unclear comprehension and negative attitudes towards the emerging infectious disease among general population may lead to unnecessary worry and even panic. The objective of this study was to investigate the Chinese public response to H1N1 pandemic and provide baseline data to develop public education campaigns in response to future outbreaks.

**Methods:**

A close-ended questionnaire developed by the Chinese Center for Disease Control and Prevention was applied to assess the knowledge, attitudes and practices (KAP) of pandemic (H1N1) 2009 among 10,669 responders recruited from seven urban and two rural areas of China sampled by using the probability proportional to size (PPS) method.

**Results:**

30.0% respondents were not clear whether food spread H1N1 virusand. 65.7% reported that the pandemic had no impact on their life. The immunization rates of the seasonal flu and H1N1vaccine were 7.5% and 10.8%, respectively. Farmers and those with lower education level were less likely to know the main transmission route (cough or talk face to face). Female and those with college and above education had higher perception of risk and more compliance with preventive behaviors. Relationships between knowledge and risk perception (OR = 1.69; 95%CI 1.54-1.86), and knowledge and practices (OR = 1.57; 95%CI 1.42-1.73) were found among the study subjects. With regard to the behavior of taking up A/H1N1 vaccination, there are several related factors found in the current study population, including the perception of life disturbed (OR = 1.29; 95%CI 1.11-1.50), the safety of A/H1N1 vaccine (OR = 0.07; 95%CI 0.04-0.11), the knowledge of free vaccination policy (OR = 7.20; 95%CI 5.91-8.78), the state's priority vaccination strategy(OR = 1.33; 95%CI 1.08-1.64), and taking up seasonal influenza vaccine behavior (OR = 4.69; 95%CI 3.53-6.23).

**Conclusions:**

This A/H1N1 epidemic has not caused public panic yet, but the knowledge of A/H1N1 in residents is not optimistic. Public education campaign may take the side effects of vaccine and the knowledge about the state's vaccination strategy into account.

## Background

At the end of March 2009, an outbreak of novel influenza A (H1N1) (here after called A/H1N1) infection occurred in Mexico, followed by ongoing spread to all over the world in a short period [1]. On June 11 2009, the World Health Organization raised its pandemic alert level to the highest level, phase 6 [2], meaning that the A/H1N1 flu had spread in more than two continents and reached pandemic proportions. As of June 13, 2010, it had caused over 18,172 deaths in more than 214 countries and overseas territories or communities [3]. Most illness, especially the severe illness and deaths, had occurred among healthy young adults, which was markedly different from the disease pattern seen during epidemics of seasonal influenza [4,5].

China is highly susceptible to A/H1N1 because of its huge population and high residential density, besides the high infectiousness of this novel influenza virus. After the first imported case reported on May 11, 2009, the confirmed cases were reported in various provinces of China [6]. By the late of October 2009, A/H1N1 cases had increased dramatically, with 44,981 cases and 6 deaths confirmed at the end of October 2009. The A/H1N1 infection rate peaked in November 2009, when approximately 1500 new cases of A/H1N1 were being confirmed each day. By the end of this month, a total of 92,904 cases and 200 deaths had resulted from A/H1N1-related causes [7]. The Chinese government has taken a series of preventive measures according to WHO guidelines, including the promotion of public knowledge about flu through mass media, patient isolation, quarantine of close contact person, and free vaccinations to population at high risk (e.g. young children, healthcare workers, and people with chronic disease) [8]. However, there were few public reports on the assessment of the effect of these policies and the level of knowledge, attitude and practice (KAP) associating with A/H1N1 among general population.

It is well-known that confused comprehension and negative attitude towards the emerging communicable disease may lead to unnecessary worry and chaos, even excessive panic which would aggravate the disease epidemic [9]. For instance, during SARS epidemic from 2002 to 2004, the misconceptions and the excessive panic of Chinese public to SARS led the public resistant to comply with the suggested preventive measures such as avoiding public transportation, going to hospital when they were sick, which contributed to the rapid spread of SARS and resulted in a more serious epidemic situation, making China one of the worst affected countries with over 5327 cases and 439 deaths [10,11]. In addition, the panic of infectious disease outbreak could cause huge economic loss, for example the economic loss of SARS has been estimated at $30-$100 billion in US, though less than 10,000 persons were infected [12]. SARS experience has demonstrated the importance of monitoring the public perception in disease epidemic control, which may affect the compliance of community to the precautionary strategies. Understanding related factors affecting people to undertake precautionary behavior may also help decision-makers take appropriate measures to promote individual or community health. Therefore, it is important to monitor and analyze the public response to the emerging disease.

To investigate community responses to A/H1N1 in China, we conducted this telephone survey to describe the knowledge, attitudes and practices of A/H1N1 among general population in China and put forward policy recommendations to government in case of future similar conditions.

## Methods

### Study sites

This study was performed in seven urban regions (Beijing, Shanghai, Wuhan, Jingzhou, Xi'an, Zhengzhou, Shenzhen cities) and two rural areas (Jingzhou and Zhengzhou counties) of China with over one million people in each region. Regarding the urban sites, Beijing as the capital of China locates in the northeast; Shanghai is a municipality in the east of China; Wuhan (the provincial capital of Hubei) and Zhengzhou (the provincial capital of Henan province) are both in the centre of China; Xi'an in the northwest of China is the provincial capital of Shanxi province; and Shenzhen of the Guangdong province is in the southeast of China. As for the rural sites, Jingzhou county and Zhengzhou county, from Hubei and Henan provinces, respectively, both locate in the centre of China.

### Sample size

This current study was carried out in three phases during the pandemic peak season of A/H1N1. The first phase was from 30 November 2009 to 27 December 2009, the second from 4 January 2010 to 24 January 2010, and the third from 24 February to 25 March in 2010.

A two-stage proportional probability to size (PPS) sampling method was used in each phase. In stage І, about 30% of administrative regions in each study site were selected as primary sample units (PSUs) for cluster sampling. In stage II, telephone numbers were sampled randomly, of which the first four digitals were obtained from each PSU's post office as initial number and the other three or four digitals were obtained from random number generated by Excel 2003. Then each family was chosen as per unit (excluding school, hotel public or cell phone *etc*.) and at least 400 families in each site at each phase were selected finally. If the family was selected repeatedly or refused to answer the questionnaire, we added one to the last digit of phone number and dial again. If the line was busy or of no response, we would dial three times and then give up this phone number if there was still no respondent.

### Telephone Survey

Anonymous telephone interviews were conducted from 6:30 pm to 10:00 pm so as to avoid over-presenting the non-work population by well-trained interviewers with Bachelor degree of Epidemiology. The Questionnaire to Survey the Level of Knowledge, Attitude and Practice in Different Stages of A/H1N1 Pandemic by Telephone was designed by the Chinese Centre for Disease Control and Prevention (China CDC, Beijing). The majority of the questions were closed-ended and variables in the questionnaire were categorical, except age. The inclusion criteria of subjects were: age≥18 and proper communication skills. There were seven questions related to the knowledge of A/H1N1, four referred to the attitude, and five concerning about the practice in this questionnaire (See additional file 1: The Questionnaire to Survey the Level of Knowledge, Attitude and Practice in Different Stages of H1N1 Pandemic by Telephone in China).

This study was approved by the institutional review board of the Tongji Medical College of Huazhong University of Science and Technology. All respondents were informed consent. We respected their wishes whether to accept our survey and promised to protect their secrets.

### Data analysis

All data were entered into computer using Epidata V.3.1 and were analyzed in SPSS statistical software V.12. Chi-square test was applied to compare the immunization rates of the seasonal flu and A/H1N1 vaccine. The associations between the socio-demographic factors and the KAP regarding A/H1N1 were firstly investigated by using univariate odds ratios (OR) and then stepwise logistic regression modeling applied. Adjusting for such background variables including gender, age, level of education, occupation, region, and survey wave, stepwise multivariate logistic regression models were applied to investigate the impact factors associated with the risk perception of A/H1N1, A/H1N1 vaccination uptake and the compliance with suggested preventive measures (avoid crowd places/wash hand frequently/keep distance from people with influenza-like symptoms). For the purposes of analysis, the factor knowledge about the main modes of transmission was divided into two groups according to whether the respondents knew both cough and talk face-to-face can spread A/H1N1. Odds ratios and respective 95% confidence intervals (CI) were obtained from the logistic regression analysis. *P *values lower than 0.05 were judged to be statistically significant.

## Results

### Socio-demographic characteristics

A total of 88541 telephone numbers were dialed. Except 65323 invalid calls (including vacant numbers, fax numbers, busy tone numbers and non-qualified respondents whose age <18 and whose phones were from school, hotel or other public places), 23218 eligible respondents were identified. Among these respondents, 12360 completed the interview. Therefore, the response rate was 46.8%. Excluding missing, and logical erroneous data, 10669 questionnaires in total were eligible for analysis. The baseline characteristics of the respondents were presented in Table1. The mean age of all respondents was 41.47 years (over range: 18-90 year). Of all respondents, 54.4% were female, and 42.4% had received college or above education (Table 1).

**Table 1:** Characteristics of the 10669 respondents, China

Characteristics		**No**.	%
Gender	Male	4864	45.6
	Female	5805	54.4
Age group (y)	18~	1446	13.5
	25~	5898	55.3
	50~	2173	20.4
	65~	1152	10.8
Occupation	Student	729	6.8
	Teacher	429	4.0
	Health care worker	338	3.2
	Office staff	2681	25.1
	Worker	1069	10.0
	Farmer	1212	11.4
	Public service personnel	848	8.0
	Others	3363	31.5
Educational background	Primary school and illiterate	1202	11.3
	Middle school	4943	46.3
	College and above	4524	42.4

### Knowledge related to the Pandemic (A/H1N1) 2009

The overall KAP related to A/H1N1 was reported in Table 2. As to knowledge, 75.6% of all respondents knew that influenza could be transmitted by coughing and sneezing, and 61.9% thought that talking face-to-face was the transmission route, whereas 30.0% believed the transmission could be through food. Less than one third of respondents knew that virus could be transmitted by handshaking and indirect hand contact (26.8% and 22.3%, respectively). Multiple logistic regression analysis showed that those with middle school (OR = 1.71; 95%CI 1.48-1.98), or having an education level of college and above (OR = 2.16; 95%CI 1.83-2.54) were more likely to know the transmission routes comparing with other people. Comparing with students, teachers (OR = 1.46; 95%CI 1.09-1.96) were more likely to answer the above questions correctly, but workers (OR = 0.77; 95%CI 0.62-0.97) and farmers (OR = 0.63; 95%CI 0.49-0.80) were less likely (present in Table 3). As time went by, the related knowledge of H1N1 among residents increased (OR = 1.20; 95%CI 1.08-1.34).More than half of the respondents knew prevention and control strategies for pandemic influenza in China, of which, 72.2% knew that A/H1N1 vaccination was free of charge and 68.2% were informed about the state's initial vaccination strategy.

**Table 2:** KAP of pandemic (H1N1)2009 among 10669 respondents, China n (%)

Questions	Yes (1)	No (2)	not clear (3)
Knowledge:			
Transmission route(1-5)			
1. Cough and sneeze	8063(75.6)	1020(9.5)	1586(14.9)
2. Face-to-face talk	6599(61.9)	2084(19.5)	1986(18.6)
3. Hand shaking or embracement	2854(26.8)	5185(48.6)	2630(24.6)
4. Indirect hand contact	2383(22.3)	5390(50.5)	2896(27.2)
5. Food	3204(30.0)	4470(41.9)	2995(28.1)
6. Know the free vaccination policy	7601(71.2)	3068(28.8)	-
7. Know the state's initial vaccination strategy	7280(68.2)	3389(31.8)	-
Attitudes:			
1. Has your daily life been disturbed by A/H1N1	3581(33.6)	7011(65.7)	77(0.7)
2. Do you worry about suffering from H1N1?	2679(25.1)*	7720(72.4)*	270(2.5)*
3.Approve of the state's initial vaccination strategy?	10248(96.1)	421(3.9)	-
4. Be afraid of H1N1 vaccine 's adverse reaction	982(9.2) ^**†**^	7460(69.9) ^**†**^	2227(20.9) ^**†**^
Practices:			
1. Have taken up seasonal influenza vaccine	797(7.5)	9872(92.5)	-
2. Have taken up influenza A (H1N1) vaccine	1150(10.8)	9519(89.2)	-
3. Avoid crowd places	4574(42.9)	5967(55.9)	128(1.2)
4. Wash hand frequently	6049(56.9)	4311(40.56)	267(2.5)
5. Keep distance from people with influenza-like symptoms	6119(57.4)	4011(37.6)	539(5.0)

*: For this question, 1 refers to no, 2 refers to a little, 3 refers to very much.

^**†**^: For this question, 1 refers to afraid, 2 refers to not afraid, 3 refers to don't care.

-: no this answer set.

**Table 3:** Associations between background characters and KAP regarding A/H1N1

	Know the main modes of transmission		worry about infection of A/H1N1		take up preventive measures		take up A/H1N1 vaccination	
				
Variables	Row%	**OR**_**m**_**(95% CI)**	Row%	**OR**_**m**_**(95% CI)**	Row%	**OR**_**m**_**(95% CI)**	Row%	**OR**_**m**_**(95% CI)**
Gender								
Male	58.5	NU	25.5	1.00	75.4	1.00	11.1	NU
Female	58.4		29.5	1.24(1.14-1.36) ^‡^	80.4	1.41(1.28-1.55) ^‡^	10.5	
Age (years)								
18~	60.5	1.00	31.1	1.00	79.1	1.00	20.5	1.00
25~	60.9	1.07(0.93-1.24)*	30.1	1.10(0.94-1.29) ^‡^	79.1	1.18(0.99-1.40)	10.7	1.15(0.91-1.46) ^†^
50~	54.4	1.04(0.88-1.24)	23.0	0.87(0.72-1.04)	75.3	1.12(0.92-1.38)	6.4	0.78(0.57-1.05)*
65~	50.5	0.87(0.72-1.06)*	19.5	0.77(0.62-0.96) ^†^	77.2	1.34(1.06-1.69)*	7.0	0.92(0.65-1.30)
Education								
Primary school and illiterate	38.0	1.00	22.7	1.00	64.6	1.00	4.7	1.00
Middle school	55.6	1.71(1.48-1.98) ^†^	26.3	1.12(0.95-1.33)	79.3	2.00(1.71-2.34) ^‡^	8.9	1.12(0.82-1.53)
College and above	66.9	2.16(1.83-2.54) ^‡^	30.4	1.23(1.02-1.49) *	80.4	1.98(1.64-2.38) ^‡^	14.5	1.09(0.78-1.52)
Occupation								
Student	62.7	1.00	33.1	1.00	80.3	1.00	35.9	1.00
Teacher	73.2	1.46(1.09-1.96) ^‡^	36.8	1.12(0.84-1.50) ^†^	82.5	0.94(0.66-1.33)	28.0	0.67(0.48-0.94) ^‡^
Health care worker	71.0	1.31(0.97-1.78) ^†^	29.6	0.74(0.54-1.02)	87.3	1.36(0.91-2.03) ^†^	46.2	1.52(1.09-2.11) ^‡^
Office staff	67.1	1.08(0.88-1.33) ^†^	29.4	0.81(0.65-1.01)	78.0	0.76(0.59-0.97) ^†^	7.9	0.13(0.10-0.17) ^‡^
Worker	54.4	0.77(0.62-0.97) ^†^	26.6	0.74(0.58-0.95)	81.0	0.99(0.74-1.31)	6.6	0.12(0.09-0.17) ^‡^
Farmer	41.3	0.63(0.49-0.80) ^‡^	25.5	0.76(0.59-0.99)	69.1	0.70(0.53-0.94) ^†^	3.1	0.06(0.04-0.10) ^‡^
Public service personnel	57.6	0.81(0.64-1.02) *	28.4	0.80(0.63-1.03)	80.3	0.89(0.67-1.18)	10.9	0.19(0.14-0.26) ^†^
Others	55.0	0.83(0.67-1.02) ^†^	24.6	0.66(0.53-0.83) ^‡^	78.1	0.74(0.57-0.96) ^†^	5.9	0.11(0.08-0.15) ^‡^
Survey wave								
30 Nov 2009-27 Dec 2009	56.9	1.00	34.3	1.00	82.6	1.00	9.1	1.00
4 Jan 2010-24 Jan 2010	57.7	1.08(0.98-1.20)	26.6	0.70(0.62-0.78)	77.7	0.91(0.80-1.03)	9.7	1.12(0.92-1.33)
24 Feb 2010-25 Mar 2010	60.6	1.20(1.08-1.34) ^†^	22.1	0.54(0.48-0.60) ^‡^	74.1	0.71(0.62-0.80) ^‡^	13.5	1.67(1.40-1.99)

OR_m_: odds ratio obtained from stepwise multivariate logistics regression analysis using univariately significant variables as candidate variables and adjusting for region;

NU: not significant in the univariate analysis;

*: *P *< 0.05;

†: *P *< 0.01;

‡: *P *< 0.0001.

### Attitudes towards the Pandemic (A/H1N1) 2009

Of all respondents, 33.6% considered that the influenza pandemic disturbing their daily lives, but did not result in public panic as 72.4% indicated they did not worry about suffering from A/H1N1. Multiple logistic regression analysis showed that females (OR = 1.24; 95%CI 1.14-1.36), people having college and above education (OR = 1.23; 95%CI 1.02-1.49), people knowing the main modes of transmission (OR = 1.69; 95%CI 1.54-1.86) and the state's initial vaccination strategy(OR = 1.12; 95%CI 1.01-1.24) were more likely to believe that they were at high risk of being infected. As time went by, the risk perception decreased (OR = 0.54; 95%CI 0.48-0.60) (in Table 3 and Table 4). Regarding the A/H1N1vaccination, 69.9% respondents believed that the occurrence rate of adverse reactions caused by A/H1N1 vaccination was fairly low and they were not afraid of taking up vaccination. Most residents (96.1%) thought that the state's vaccination strategy was reasonable.

**Table 4:** Factors associated with the risk perception of A/H1N1 or taking up preventive measures in daily life

	Worry about infection of A/H1N1			Take up preventive measures in daily life		
		
Variables	Row%	**OR**_**u **_**(95% CI)**	**OR**_**m**_**(95% CI)**	Row%	**OR**_**u **_**(95% CI)**	**OR**_**m**_**(95% CI)**
Know the main modes of transmission						
No	21.8	1.00	1.00	71.9	1.00	1.00
Yes	31.8	1.68(1.53-1.83) ^†^	1.69(1.54-1.86) ^†^	82.5	1.84(1.68-2.02) ^†^	1.57(1.42-1.73) ^†^
Know the state's initial vaccination strategy						
No	24.9	1.00	1.00	68.3	1.00	1.00
Yes	28.9	1.22(1.12-1.34) ^†^	1.12(1.01-1.24)*	82.7	2.21(2.02-2.43) ^†^	1.61(1.43-1.81) ^†^
Know the free vaccination policy						
No	26.5	1.00	NU	69.4	1.00	1.00
Yes	28.1	1.08(0.98-1.19)		81.6	1.96(1.78-2.16) ^†^	1.40(1.24-1.58) ^†^
Worry about infection of A/H1N1						
No	-	-	-	74.4	1.00	1.00
Yes				87.9	2.51(2.22-2.84) ^†^	2.09(1.84-2.37) ^†^
Has your daily life been disturbed by A/H1N1						
No	-	-	-	73.4	1.00	NS
Yes				87.2	2.47(2.22-2.76) ^†^	

OR_u_: univariate odds ratio obtained using logistic regression;

OR_m_: odds ratio obtained from stepwise multivariate logistics regression analysis using univariately significant variables as candidate variables and adjusting for gender, age, education level, occupation, region and survey wave;

NU: not significant in the univariate analysis;

NS: not significant in multivariate analysis though significant in the univariate analysis;

-: not analyzed

*: *P *< 0.05;

†: *P *< 0.0001.

### Practices related to the Pandemic (A/H1N1) 2009

About half of the respondents (42.9%) had avoided going to crowded places during the past two weeks of our survey. In case people nearby held influenza-like symptoms such as fever or cough, 56.9% increased the frequency of hand-washing and 57.4% would stay away from them. Multiple logistic regression analysis indicated compliance with the preventive practices were more likely to be taken by those who were females (OR = 1.41; 95%CI 1.28-1.55), old people above 65 years old (OR = 1.34; 95%CI 1.06-1.69), people with education level middle school (OR = 2.00; 95%CI 1.71-2.34) or College and above (OR = 1.98; 95%CI 1.64-2.38), people perceiving a higher risk of being infected (OR = 2.09; 95%CI 1.84-2.37) and those who knew the main modes of transmission (OR = 1.57; 95%CI 1.42-1.73), but less likely to be taken by staff working in office (OR = 0.76; 95%CI 0.59-0.97) and farmers (OR = 0.70; 95%CI 0.53-0.94) comparing with students. As time went by, public became less likely to take the preventive practices (OR = 0.70; 95%CI 0.53-0.94). (in Table 3 and Table 4).

The immunization rates of the seasonal flu and A/H1N1 in respondents were 7.5% and 10.8% respectively. The multivariate stepwise models further showed that except the health care workers (OR = 1.52; 95%CI 1.09-2.11), residents in other occupations (OR = 0.06-0.67) were less likely to take up the A/H1N1 vaccination comparing with students (in Table 3). Adjusting for the background covariates the knowledge about the free vaccination policy (OR = 7.20; 95%CI 5.91-8.78) and the state's initial vaccination strategy(OR = 1.33; 95%CI 1.08-1.64), perception of daily life disturbed (OR = 1.29; 95%CI 1.11-1.50), practice of injecting the seasonal influenza vaccine (OR = 4.69; 95%CI 3.53-6.23) were significantly associated with behavior of taking up the A/H1N1 vaccination positively (in Table 5), and the adverse reaction of A/H1N1 vaccine negatively influenced people's practice (OR = 0.07; 95%CI 0.04-0.11).

**Table 5:** Factors associated with A/H1N1 vaccination uptake

Variables	Vaccinated rate(%)	**OR**_**u **_**(95% CI)**	**OR**_**m**_**(95% CI)**
Know the main modes of transmission			
No	8.4	1.00	NS
Yes	12.5	1.56(1.37-1.78) ^‡^	
Know the free vaccination policy			
No	2.3	1.00	1.00
Yes	14.2	8.71(7.44-10.20) ^‡^	7.20(5.91-8.78) ^‡^
Know the state's initial vaccination strategy			
No	4.7	1.00	1.00
Yes	13.6	3.18(2.67-3.77) ^‡^	1.33(1.08-1.64) ^†^
Has your daily life been disturbed by A/H1N1			
No	9.1	1.00	1.00
Yes	14.0	1.63(1.44-1.84) ^‡^	1.29(1.11-1.50) ^‡^
Would worry about infection of A/H1N1			
No	10.5	1.00	NU
Yes	11.6	1.12 (0.98-1.28)	
Is the state's initial vaccination strategy reasonable			
No	7.6	1.00	NS
Yes	10.9	1.49(1.03-2.15) *	
The reaction to the adverse reaction of A/H1N1 vaccine			
Not afraid	14.8	1.00	1.00
Don't care	1.4	0.08(0.06-0.11)	0.08(0.05-0.11) ^‡^
Be afraid	1.8	0.11(0.07-0.17) ^†^	0.07(0.04-0.11) ^‡^
Have taken up preventive measures in daily life			
No	7.5	1.00	NS
Yes	11.7	1.64(1.38-1.93) ^‡^	
Have taken up seasonal influenza vaccine			
No	8.1	1.00	1.00
Yes	43.5	7.09(5.55-9.07) ^‡^	4.69(3.53-6.23) ^‡^

OR_u_: univariate odds ratio obtained using logistic regression;

OR_m_: odds ratio obtained from stepwise multivariate logistics regression analysis using univariately significant variables as candidate variables and adjusting for gender, age, education level, occupation, region and survey wave;

NU: not significant in the univariate analysis;

NS: not significant in multivariate analysis though significant in the univariate analysis;

*: *P *< 0.05;

†: *P *< 0.01;

‡: *P *< 0.0001.

## Discussion

Novel A/H1N1 has caused pandemic in this century. It is important to encourage the public to adopt precautionary behaviors, which is based on the correct knowledge of the epidemic and appropriate response among residents. Many studies have examined the various levels of KAP about infectious disease outbreaks, such as SARS, avian influenza [13-15]. Some studies have been reported specifically on community responses to A/H1N1 in Australia and Europe [16,17]. But through literature search, we haven't found any public reports on KAP regarding A/H1N1 among Chinese population until now. Therefore, we conducted this large population-based survey (10669 respondents) to investigate community responses to A/H1N1 and to provide baseline data to government for preventive measures in case of future outbreaks.

Unless people have basic knowledge about the modes of transmission, they respond appropriately during an outbreak [16]. It has been proved that influenza is transmitted through person to person via respiratory secretions [18]. Most residents in our survey recognized that the risk of getting infected would increase when an infected person coughed or sneezed in close distance. This may be due to the previous experience of SARS and avian flu. Multivariate analysis results showed that workers and farmers with lower education level were less likely to have this knowledge, which indicated that the contents and forms of propaganda should be more understandable and acceptable. A large proportion of residents in our survey overlooked the indirect hand contact and hand-shaking transmission route and about one third of public misconceived that A/H1N1 was food borne, which was associated with the previous knowledge of avian flu and the new A/H1N1 flu in the general population. The confusion with avian flu might mislead some residents to believe that the A/H1N1 virus is fatal and cause public panic [19]. Therefore, it is important for the government and health authorities to provide continuously updated information of the emerging disease through televisions, newspapers, radios, and Internet.

There are regional differences in the perception of A/H1N1. For example, the public in Hong Kong did not perceive a high likelihood of having a local A/H1N1 outbreak [19], but Malaysians were particularly anxious about the pandemic [20]. The current study shows that emotional distress was relatively mild in China as few residents worried about being infected (25.1%). This phenomenon may also be related to the previous experience of the SARS epidemic, as well as the open epidemic information. A survey in Korean university showed that women perceived higher illness severity and personal susceptibility to A/H1N1 infection, which had been reconfirmed in our study [21]. Logistic regression analysis results suggested that women with higher educational level had higher perception of risk. As time went by, the knowledge about the main transmission route increased, but the risk perception of being infected in residents decreased, suggesting the positive effect of government policy regarding A/H1N1 infection prevention, as well as the promotion of the media.

The previous study presented various results of influencing factors on the the compliance with the preventive practices. The study in Saudi showed that older men with better education were more likely to take preventive practices [9]; female students in Korean washed hands more frequently during the peak pandemic period of A/H1N1 [21]; in another pandemic study in USA, younger people was found to have greater uptake of recommended behaviors but not for gender [16]. We found female with higher education took more precautionary behaviors, but office staffs and farmers took less comparing with students. While such differences could result from study population demographics, profound differences may also exist in the knowledge of A/H1N1 and the perceptions of recommended behaviors in those countries. Adjusting for the background factors, the multivariate logistic regression showed the possible relationship between knowledge and risk perception, knowledge and practices (odd ratios were 1.57 and 2.09, respectively), which indicated that good knowledge is important to enable individuals to have better attitudes and practices in influenza risk reduction. Similar findings were observed in other studies performed during A/H1N1 pandemic in Singapore [22] and during SARS pandemic in Hong Kong [13]. Therefore, it is important to focus on inculcating the correct knowledge to individuals as it will influence both attitudes and practices.

Injecting vaccination is an effective measure to prevent infectious disease [23]. In China, the seasonal influenza vaccination is not included in the national immunization program and must be purchased by recipients. Those who are above 60 years old, the pupil and children in kindergarten, and people with chronic diseases are recommended to get inoculation. Data provided by China CDC in 2009 showed that the immunization rate of the seasonal flu in Chinese population was below 2% [24], which was much lower than 7.5% in our study (*P *< 0.0001). This phenomenon is partly due to the state's prior vaccination strategy for population at high risk such as students, teachers, healthcare workers and people with chronic disease, as well as the confusion between seasonal flu vaccine and A/H1N1 vaccine in residents. People who couldn't access the A/H1N1 vaccine may take up seasonal flu vaccine as preventive behaviors. The A/H1N1 vaccine was not available in China until the middle of September 2009. All populations at high risk above three years old were invited for vaccination free of charge [25]. A survey among 868 European travelers showed 14.2% participants were vaccinated against pandemic influenza A/H1N1 [26], higher than 10.8% in our study (*P *< 0.01). Our study also showed students and health care workers were more likely to take up, which may be due to the prior vaccination strategy. Multivariate stepwise logistic regression analysis, which allowed us to adjust for background factors, further showed the perceived risk of infection and the knowledge about the main modes of transmission related to A/H1N1 vaccination were insignificantly, similar results seen in Lau's study[8]. Therefore, the vaccination rate of A/H1N1 is not expected to increase even if the virus becomes more prevalent or the knowledge of its transmission mode improved. Additionally, the behavior of taking up A/H1N1 vaccine was associated with perceptions of vaccine's safety and influence on daily life by A/H1N1 as well as the knowledge about the free vaccination policy and the state's initial vaccination strategy. This suggests that improving the safety of vaccine, the acceptability of side effect and the knowledge about the state's strategy related to A/H1N1 vaccination in residents may be helpful to promote A/H1N1 vaccination in the general population.

The cross-sectional telephone survey adopted in the study has some limitations. We were unable to interview the people who did not have phones and the depth of the questionnaire was largely limited because questions and pre-existing answers could not be too long and complex. In addition, the telephone response rate was 46.8%, which means more than half of the interviewees rejected or didn't finish the survey. It was impossible to compare the difference between respondents and non-respondents due to the lack of their basic information.

## Conclusions

This A/H1N1 epidemic has not caused public panic yet, but the knowledge of A/H1N1 in residents is not optimistic as most of them confused the transmission route of A/H1N1. There are many factors influencing the KAP related to A/H1N1. Female with higher educational level had higher perceived risk of infection and took more precautionary behaviors. Public education campaign may take the side effects of vaccine and the knowledge about the state's vaccination strategy into account. The data collected in this survey could be used as baseline data to monitor public perceives and behaviors in the event of future outbreak of infectious disease in China.

## Competing interests

The authors declare that they have no competing interests.

## Authors' contributions

HY and YX participated in design and coordination, and helped to draft the manuscript. YL and LH participated in the telephone investigations and analyzed the data and drafted the manuscript. SN conceived of the study, participated in its design and coordination. ZL participated in collection and management of data. WY participated in design and coordination, and helped to revise the manuscript. All authors read and approved the final manuscript.

## Pre-publication history

The pre-publication history for this paper can be accessed here:

http://www.biomedcentral.com/1471-2334/11/128/prepub

## Supplementary Material

Additional file 1 **Questionnaire**. The Questionnaire to Survey the Level of Knowledge, Attitude and Practice in Different Stages of H1N1 Pandemic by Telephone in China. Click here for file
