# Supplementary material for: Knowledge, Attitudes and Practices (KAP) related to the Pandemic (H1N1) 2009 among Chinese General Population: a Telephone Survey
Source: BMC Infect Dis. 2011 May 16;11:128. doi: 10.1186/1471-2334-11-128 (PMC3112099; doi:10.1186/1471-2334-11-128)
Supplement: Additional file 1 — Questionnaire. The Questionnaire to Survey the Level of Knowledge, Attitude and Practice in Different Stages of H1N1 Pandemic by Telephone in China. [file 1471-2334-11-128-S1.PDF]

The Questionnaire to Survey the Level of Knowledge, Attitudes and Practices (KAPs) in Different Stages of H1N1 Pandemic by Telephone in China.

Introduction

After the first imported case reported on May 11, 2009 of Influenza A (H1N1), the confirmed cases were consecutively reported in various provinces of China. China is highly susceptible to H1N1 because of its huge population and high residential density, besides the high infectiousness of this novel influenza. A telephone survey is planed to carry out to investigate community responses to H1N1, to describe the knowledge, attitudes and practices of H1N1 among Chinese residents and put forward policy recommendations to government in case of future outbreaks. We thank you for your participation in our investigation with contribution to fill out a questionnaire in the next page.

This questionnaire will investigate your knowledge, attitudes and practices (KAP) to seasonal influenza and influenza A (H1N1). Some questions can simply be answered by yes or no. Please do not take too much time to answer each question and do not consult with your colleagues.

In case you doubt about the answer to be given, try to choose the possibility that nears best reality. Do never mark "yes" and "no" at the same time, as in that case your answer can no longer be processed.

Your answers will be treated in the strictest confidence. Apart from the research workers no one will ever have access to the data without your permission, not even your superiors. In the report about this study your personal data cannot be recognized.

Thank you again for your cooperation.

ID: □□□□□□□ **Phone Number**： □□□□—□□□□□□□□

First Part: General questions

1. What is your age? ___ years

2. What is your gender?

(1) male (2)female

3. What is the **highest** education that you completed successfully?

(1)no education completed or primary school (2)Middle school (3) College and above

4. What is your occupation?

(1) Student (2) Teacher (3) Healthcare worker (4) Office staff (5) Worker (6) Public service personnel （7）Farmer （8）Others

Second Part: Knowledge to Influenza A (H1N1):

1. Do you think H1N1 can be transmitted by cough or sneeze?

(1) Yes (2) No (3) Not clear

2. Do you think H1N1 can be transmitted by face-to-face talk?

(1) Yes (2) No (3) Not clear

3. Do you think H1N1 can be transmitted by handing shaking or embracement?

(1) Yes (2) No (3) Not clear

4. Do you think H1N1 can be transmitted by indirect hand contact?

(1) Yes (2) No (3) Not clear

5. Do you think H1N1 can be transmitted by food?

(1) Yes (2) No (3) Not clear

6. Do you know the free vaccination policy of H1N1?

(1) Yes (2) No

7. Do you know the state's initial vaccination strategy for population at high risk including young children, healthcare workers, public health workers and people with chronic disease?

(1) Yes (2) No

Third Part: Attitude to Influenza A (H1N1):

1. Has your daily life been disturbed by A/H1N1?

(1)Yes (2)No (3)Not clear

2. Do you worry about suffering from H1N1?

(1)No, Never or the same with usual (2) Yes, a little (3) Yes, very much

3. Do you think the state's initial vaccination strategy for population at high risk including young children, medical staff, public health workers and people with chronic disease reasonable?

(1)Yes (2)No

4. What's your opinion to the adverse reaction of H1N1 vaccine?

(1) Afraid (2) Not afraid (3) Don't care

Fourth Part: Practice to Influenza A (H1N1):

1. Have you taken up seasonal influenza vaccine since July, 2009?

(1)Yes (2)No

2. Have you taken up influenza A (H1N1) vaccine?

(1)Yes (2)No

3. Have you taken measures such as avoiding going to crowd places in the last two weeks?

(1)Yes (2)No (3)Not clear

If someone nearby got influenza-like symptoms such as fever or cough

4. Have you taken measures such as increasing the frequency of hand-washing?

(1)Yes (2)No (3)Not clear

5. Have you taken measures such as keeping away from the people?

(1)Yes (2)No (3)Not clear

Fifth Part: General evaluation

1. Evaluation of respondent’s cooperation degree: (1)better (2)good (3) bad

2. Evaluation of information’s credibility degree: (1)better (2)good (3) bad

Investigator’s signature: _____________ Investigate Date: ___________________

Review staff’ signature: _____________ Investigate Date: ___________________
